# sRNA EsrE Is Transcriptionally Regulated by the Ferric Uptake Regulator Fur in *Escherichia coli*

**DOI:** 10.4014/jmb.1907.07026

**Published:** 2019-11-06

**Authors:** Bingbing Hou, Xichen Yang, Hui Xia, Haizhen Wu, Jiang Ye, Huizhan Zhang

**Affiliations:** 1 State Key Laboratory of Bioreactor Engineering, East China University of Science and Technology, Shanghai, P.R. China; 2 Department of Applied Biology, East China University of Science and Technology, Shanghai, P.R. China

**Keywords:** Small RNA, EsrE, DNA affinity chromatography, Fur, transcriptional regulation

## Abstract

Small RNAs (sRNAs) are widespread and play major roles in regulation circuits in bacteria. Previously, we have demonstrated that transcription of *esrE* is under the control of its own promoter. However, the regulatory elements involved in EsrE sRNA expression are still unknown. In this study, we found that different *cis*-regulatory elements exist in the promoter region of *esrE*. We then screened and analyzed seven potential corresponding *trans*-regulatory elements by using pull-down assays based on DNA affinity chromatography. Among these candidate regulators, we investigated the relationship between the ferric uptake regulator (Fur) and the EsrE sRNA. Electrophoresis mobility shift assays (EMSAs) and β-galactosidase activity assays demonstrated that Fur can bind to the promoter region of *esrE*, and positively regulate EsrE sRNA expression in the presence of Fe^2+^.

## Introduction

Bacteria can effectively respond to external environments and stresses through complex regulatory networks. Among such networks, regulatory sRNAs, ranging from 50-250 nucleotides, are widespread and play major roles in regulating translation of related mRNAs participating in biofilm formation, resistance stress, nutrient utilization, and so on [1-3]. Although the sequences of most sRNAs are not conserved among species, sRNAs are a universal phenomenon for genetic regulation in all bacteria [4]. Most known sRNAs function by forming base-pairing with the target mRNAs, affecting translation, stability, or processing of target mRNAs, thereby regulating expression of target genes [5-7]. Previous studies demonstrated that, besides the intergenic regions, sRNAs can also be derived from 5’ untranslated regions (UTRs) [8, 9], 3’ UTRs [10-12], intergenic regions [13], and antisense to coding regions [14, 15]. On the other hand, sRNA can not only be originated by processing of mRNAs [10, 16], but also be transcribed by independent promoters that are located in intergenic regions [17-19] or embedded in mRNA coding regions [20].

Typically, the expressions of sRNAs are regulated by diverse transcriptional regulators, which can directly sense biological signals or environmental changes [21]. RyhB is a well-studied sRNA existing in many bacteria, and functions as both a repressor and activator [22, 23]. Transcription of RyhB sRNA is repressed by an Fe^2+^-dependent regulator Fur, while translation of the upstream of the Fur translated region is downregulated by RyhB sRNA, making a feedback loop [24]. s-SodF is a short 3’-UTR processing product from *sodF* mRNA, which binds to *sodN* mRNA and causes its degradation. When nickel is sufficient, a Fur-family regulator Nur represses transcription of the *sodF* gene, resulting in a significant decrease of s-SodF sRNA [25]. FnrS is a highly conserved sRNA in various enterobacteria, and negatively regulates numerous mRNAs encoding enzymes involved in energy metabolism. Expression of FnrS is activated by two transcriptional regulators, FNR (fumarate and nitrate reduction) and ArcA (aerobic respiratory control), under anaerobic conditions [26, 27].

In our previous studies, we found a novel sRNA EsrE affects cell growth in *E. coli* [28]. Further studies demonstrated that EsrE is an independent transcript under the control of a promoter within the coding region of *ubiJ* (also known as *yigP*), and regulates multiple mRNAs that are involved in murein biosynthesis and the tricarboxylic acid cycle [29]. However, the relative transcriptional regulators and regulatory mechanisms of EsrE expression are still unknown. In this study, we demonstrated that Fur can bind to the promoter region of *esrE*, and positively regulate expression of EsrE sRNA in the presence of Fe^2+^.

## Material and Methods

### Bacterial Strains, Plasmids and Growth Conditions

The bacterial strains and plasmids used in this study are listed in Table 1. *E. coli* JM83 and *E. coli* BL21 (DE3) strains were used for routine molecular cloning and protein overexpression, respectively. Unless otherwise stated, the strains were routinely grown at 37 °C in liquid or solid Luria-Bertani (LB) medium supplemented with ampicillin (100 μg/ml), kanamycin (50 μg/ml), or chloramphenicol (30 μg/ml), as appropriate.

### Construction of *lacZ* Reporter Plasmids

The promoter-probe plasmid pSP-Z was used to construct reporter plasmids in this work, and was derived from pSPORT1 [30], carrying a 3.1 kb PstI/BamHI fragment containing the *lacZ* gene as a reporter. Promoter fragments, S1V3, P40V3, P42V3, and P43V3, were amplified using primer pairs S1/V3, P40/V3, P42/V3, and P43/V3 (Table 2) with genomic DNA from *E. coli* JM83 as templates, respectively. The purified fragments were digested with HindIII and NcoI, and ligated into the HindIII/NcoI-restricted pSP-Z vector, resulting in reporter plasmids pSZ1, pPZ40, pPZ42, and pPZ43, where the *lacZ* gene is under the control of a series of promoter fragments of different lengths.

### β-Galactosidase Activity Assays

The reporter plasmids were introduced into *E. coli* wild-type strain JM83 or mutant Δ*fur* as appropriate. β-galactosidase assays were performed as described in our previous work [31]. *E. coli* strains were cultivated in liquid LB medium at 37°C until reaching an optical density at 600 nm of approximately 2. Cells (3OD for each strain) were harvested and suspended in 300 μl of 100 mM phosphate-buffered saline (PBS) buffer. Then, the cells were lysed by sonication for 3 min and centrifuged for 30 min at 4°C to remove cellular debris, resulting in cell extracts. The reaction mixture included 3 μl of 100 × MgCl_2_ solution (20 μl of 1 M MgCl_2_, 63 μl of 14.3 M β-mercaptoethanol, 117 μl of ddH_2_O), 66 μl of substrate solution (60 mM Na_2_HPO_4_, 40 mM NaH_2_PO_4_, 1 mg/ml *o*-nitro-phenyl-β-D-galactopyranoside, and 2.7 μl/ml β-mercaptoethanol), 131 μl of 100 mM PBS, and 100 μl of cell extract. After 30 min at 30°C, reactions were terminated by adding 500 μl of 1M Na_2_CO_3_. The optical density at 420 nm was detected, and the enzyme activities were calculated as the change per minute per OD unit of culture present in the assays and converted into Miller units.

### DNA Affinity Chromatography

DNA affinity chromatography was performed as previously described [32] with the following modifications. DNA probes with the biotin at the 5’ end, biotin-S1V3 and biotin-ORF (negative control), were generated by PCR using primer pairs PD*-1/PD-2 and DZ*-1/DZ-2 (Table 2), respectively. The concentration and quality of the probes were analyzed by using a spectrophotometer NanoDrop 2000. Total proteins were obtained as outlined above (β-galactosidase activity assays), analyzed using 12% SDS-polyacrylamide gel electrophoresis (SDS-PAGE), and were quantified through the Bradford assay [33].

DNA probes were immobilized to M-280 Dynabeads (Invitrogen, USA) using the method recommended by the manufacturer. Briefly, streptavidin Dynabeads were washed three times and suspended in buffer A (50 mM Tris-HCl pH 7.5, 0.5 mM EDTA, 1 mM DTT, and 1 M NaCl). The DNA probes were incubated with the beads for 30 min at room temperature. Subsequently, buffer A and protein-binding buffer B (20 mM Tris-HCl pH 8.0, 1 mM EDTA, 1 mM DTT, 100 mM NaCl, and 10% glycerol) were successively used to wash the DNA-bead complex for three times. After incubation with the cell extracts for 1 h at room temperature with shaking at 350 rpm, the magnetic particles were washed three times with buffer B to remove unbound proteins. The DNA-binding proteins were eluted by elution buffer C with different concentrations of NaCl (25 mM Tris-HCl pH 8.0 and 200 mM/ 500 mM/1 M NaCl), and finally DNase I was added to the reaction mixture. The eluted fractions were subjected to SDS-PAGE, and visualized using silver staining.

The silver staining method is sensitive for visualizing low-abundance proteins [34]. The gel was cleaned with deionized water for 5-10 min, and fixed in fixative buffer (30% ethanol and 10% acetic acid) for at least 2 h. After washing with 10% ethanol solution twice, the gel was sensitized in 0.02% sodium thiosulphate solution for 1 min, followed by incubation in 0.1% (w/v) silver nitrate solution for 20 min. Subsequently, the gel was briefly washed twice with deionized water and immersed in developing solution (2% Na2CO3 and 0.04% formaldehyde) until the protein bands could be observed clearly. Finally, the reaction was were terminated by adding 5% acetic acid solution. The protein bands of interest were excised from the gel with a scalpel, and sent to Applied Protein Technology (ATP, China) for trypsin digestion and mass spectrometry.

### Construction, Overexpression and Purification of Fur

The *fur* gene was amplified by PCR using the primer pairs Fur-1/2 in Table 2, and inserted into the pET-28a (+) vector after digestion with *Nde*I/*Eco*RI, resulting in expression plasmids pFUR. The obtained plasmid was transformed into *E. coli* BL21 (DE3) for protein expression. The strain was cultivated at 37ºC until the OD_600_ reached about 0.6, and IPTG was added to a final concentration of 0.5 mM. Subsequently, the culture was incubated at 16ºC overnight.

The cell pellets were collected by centrifugation, washed twice with phosphate buffer, and re-suspended in the same buffer. The proteins were released by sonication on ice, and purified using Ni-iminodiacetic acid agarose chromatography (WeiShiBoHui, China). The purified protein was desalted by molecular exclusion chromatography using G25. The purified Fur protein was analyzed using 12% SDS-PAGE, and quantified using the Bradford assay.

### Electrophoresis Mobility Shift Assays (EMSA)

DNA probe Biotin-S1V3 was amplified by PCR using primer pairs PD*-1/PD-2, and an unlabeled DNA fragment was amplified by PCR using primer pairs PD-1/PD-2 (Table 2). EMSAs were carried out as described in our previous work [35], using chemiluminescent EMSA kits (Beyotime Biotechnology, China). The binding reaction mixture contained 10 mM Tris-HCl pH 7.5, 50 mM KCl, 0.5 mM DTT, 0.05 mg/ml BSA, 4% glycerin, 1 mM MgCl_2_, 0.1 mM MnCl_2_, 10 ng of DNA probe, 50 μg/ml poly(dI-dC), and appropriate His_6_-Fur protein.

### Inactivation of the *fur* Gene

The λ Red-mediated recombination method was used to construct a *fur* inactivation strain [36]. A 1.4 kb disruption fragment containing the apramycin resistance gene was amplified using primer pairs fur-QC1/QC2, each of which has a 5’ sequence (39 nt) matching the JM83 sequence adjacent to the *fur* gene to be inactivated. The disruption fragment was transferred to *E. coli* JM83 by electroporation. The internal region of the *fur* gene was replaced with apramycin resistance gene by λ Red-mediated recombination, to obtain the *fur* inactivation strain, which was identified by PCR using the primer pairs fur-JD1/JD2.

## Results

### The Promoter Region of *esrE* Contains Several *cis*-Regulatory Elements

**Fig. 1:**
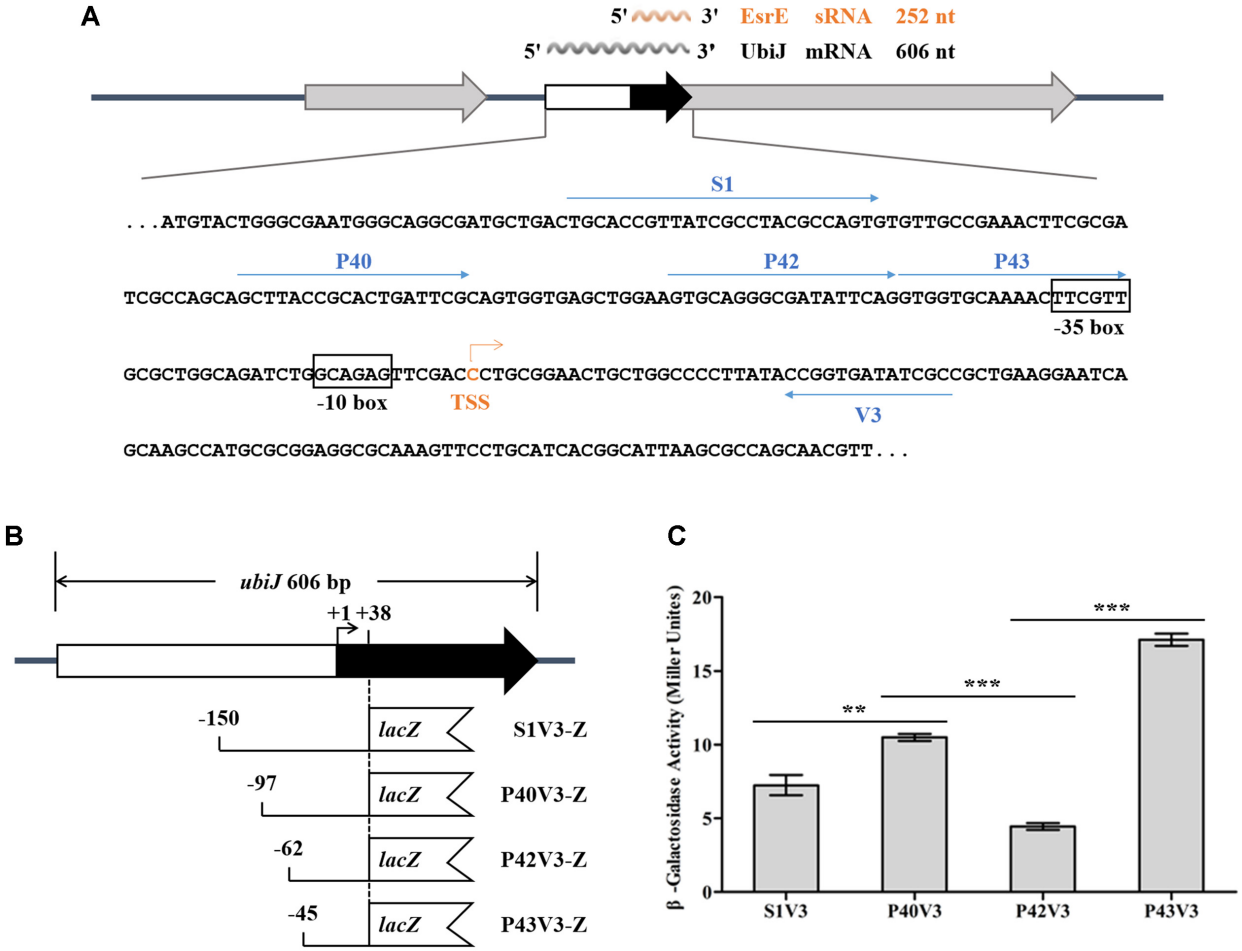
Analysis and identification of the *cis*-regulatory elements within the promoter region of *esrE*. (**A**) Schematic representation and sequence analysis of the *esrE* gene and its promoter region. The -35 and -10 boxes are indicated by hollow boxes, and the transcription start site (TSS) is indicated by an orange arrowhead. Primers (S1, P40, P42, P43, and V3) are indicated by horizontal arrows. (**B**) Locations of different length fragments in the upstream region flanking the *esrE* gene. (**C**) Basal transcriptional activities of *esrE* promoter fragments of various lengths in *E. coli* JM83. Bars correspond to the mean ± SD of three biological replicates. **p < 0.01, ***p < 0.001.

In our previous study, we have demonstrated that the *esrE* gene encodes a non-coding sRNA, which is controlled by its own promoter [28]. To further investigate the relevant regulation of EsrE expression, we analyzed the *cis*-regulatory elements upstream of the *esrE* gene. A series of fragments, S1V3, P40V3, P42V3 and P43V3, were amplified and fused to the *lacZ* reporter gene to carry out β-galactosidase activity assays (Figs. 1A and 1B). The data showed that β-galactosidase activity driven by P42V3 was significantly decreased compared to that driven by P43V3 and P40V3, suggesting the fragment P42-P43 contains negative regulatory element(s), and the fragment P40-P42 contains positive regulatory element(s) (Fig. 1C). Moreover, the β-galactosidase activity driven by S1V3 was decreased compared to that drove by P40V3, suggesting the fragment S1-P40 contains negative regulatory element(s) as well (Fig. 1C). Thus, we demonstrated that several *cis*-regulatory elements exist upstream of the *esrE* gene.

### Corresponding *Trans*-Regulatory Elements Are Identified by Pull Down

**Fig. 2:**
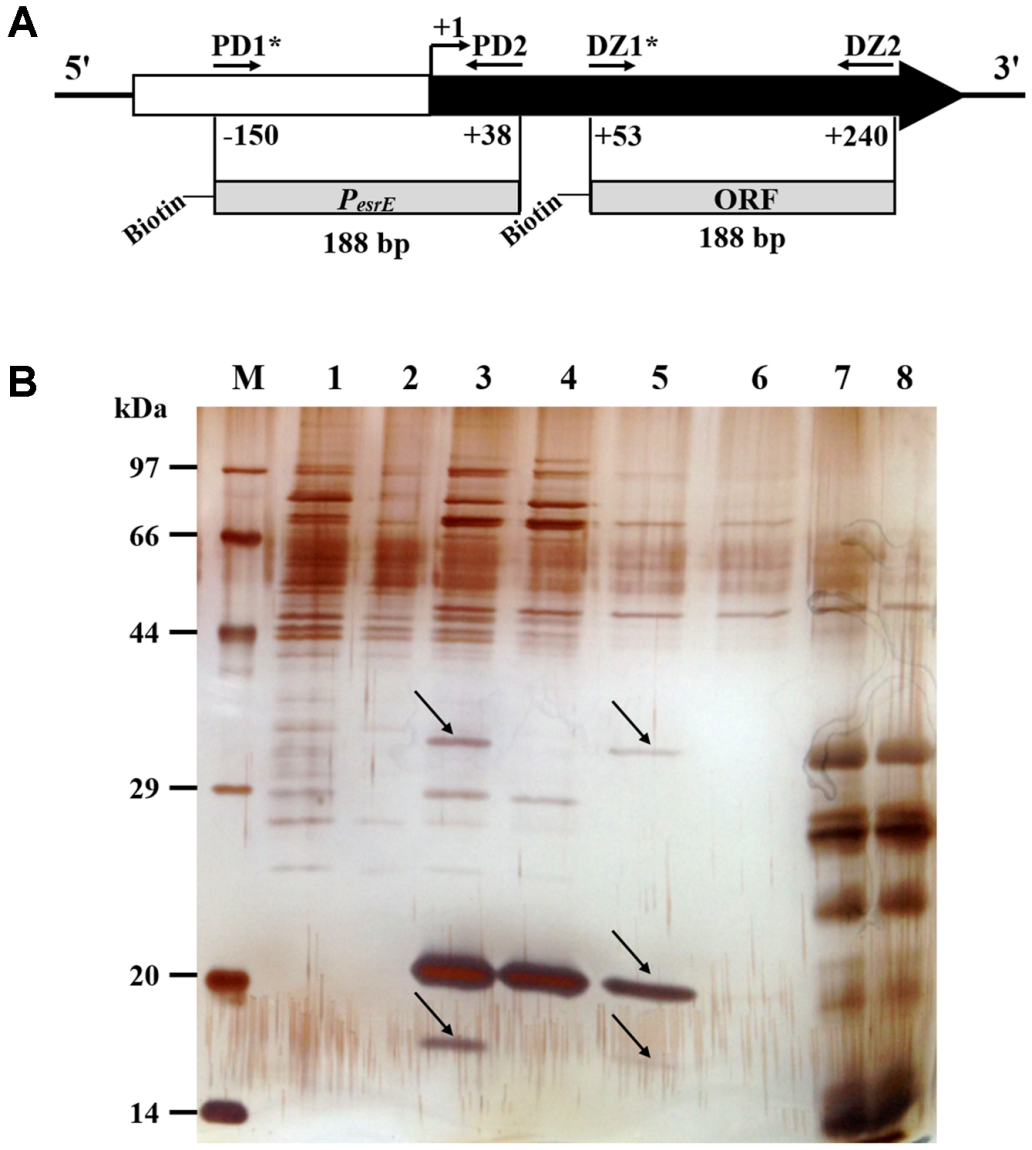
Screening for P_*esrE*_ binding proteins. (**A**) Locations of the DNA probe Biotin-P_*esrE*_ and Biotin-ORF. The primer pairs PD1*/2 and DZ1*/2 are indicated by horizontal arrows. +1 indicates the TSS of *esrE*. Asterisks indicate biotin-labeled. (**B**) SDS-PAGE of proteins analyzed through DNA affinity chromatography using Biotin-P_*esrE*_ as bait and Biotin-ORF as a negative control. Proteins that bound to the Biotin-P_*esrE*_ probe (lanes 1, 3, 5, and 7), and the Biotin-ORF fragment (lanes 2, 4, 6, and 8) were separated by SDSPAGE and visualized through silver staining. Proteins that specifically bound to the Biotin-P_*esrE*_ probe are indicated by arrows. Lane M, protein molecular weight marker. Lanes 1 and 2, eluate by 200 mM NaCl; Lanes 3 and 4, eluate by 500 mM NaCl; Lanes 5 and 6, eluate by 1 M NaCl; Lanes 7 and 8, eluate treated with DNase I.

To identify the corresponding *trans*-regulatory elements involved in transcription of the *esrE* gene, proteins binding to the promoter region of *esrE* were screened by pull-down assays based on DNA affinity chromatography. A biotin-labeled DNA probe S1V3 (designated Biotin-P_*esrE*_, 188 bp) was used as bait, and another biotin-labeled DNA fragment within the *esrE* coding region (designated Biotin-ORF, 188 bp) was used as a negative control (Fig. 2A). The affinity captured proteins were analyzed using SDS-PAGE, and visualized by silver staining. The results showed that five distinguishing protein bands with molecular weights from 14 kDa to 44 kDa were observed in lines 3 and 5 for the Biotin-P_*esrE*_ probe, compared to Biotin-ORF (Fig. 2B, arrow indicated).

Subsequently, the five selected protein bands were excised from the gel and subjected to tryptic digestion together. To identify the candidate proteins, MALDI-TOF and a peptide fingerprint analysis were performed, and the obtained peptide mass patterns were compared with the proteome of *E. coli* K-12 using MASCOT 2.2 software [37]. As a result, 7 potential DNA-binding proteins were identified excluding the ribosomal proteins and the contaminant proteins with incorrect, higher molecular weight (Tables S1 and 3).

### Fur Functions as a P_*esrE*_-Interactive Regulator

**Fig. 3:**
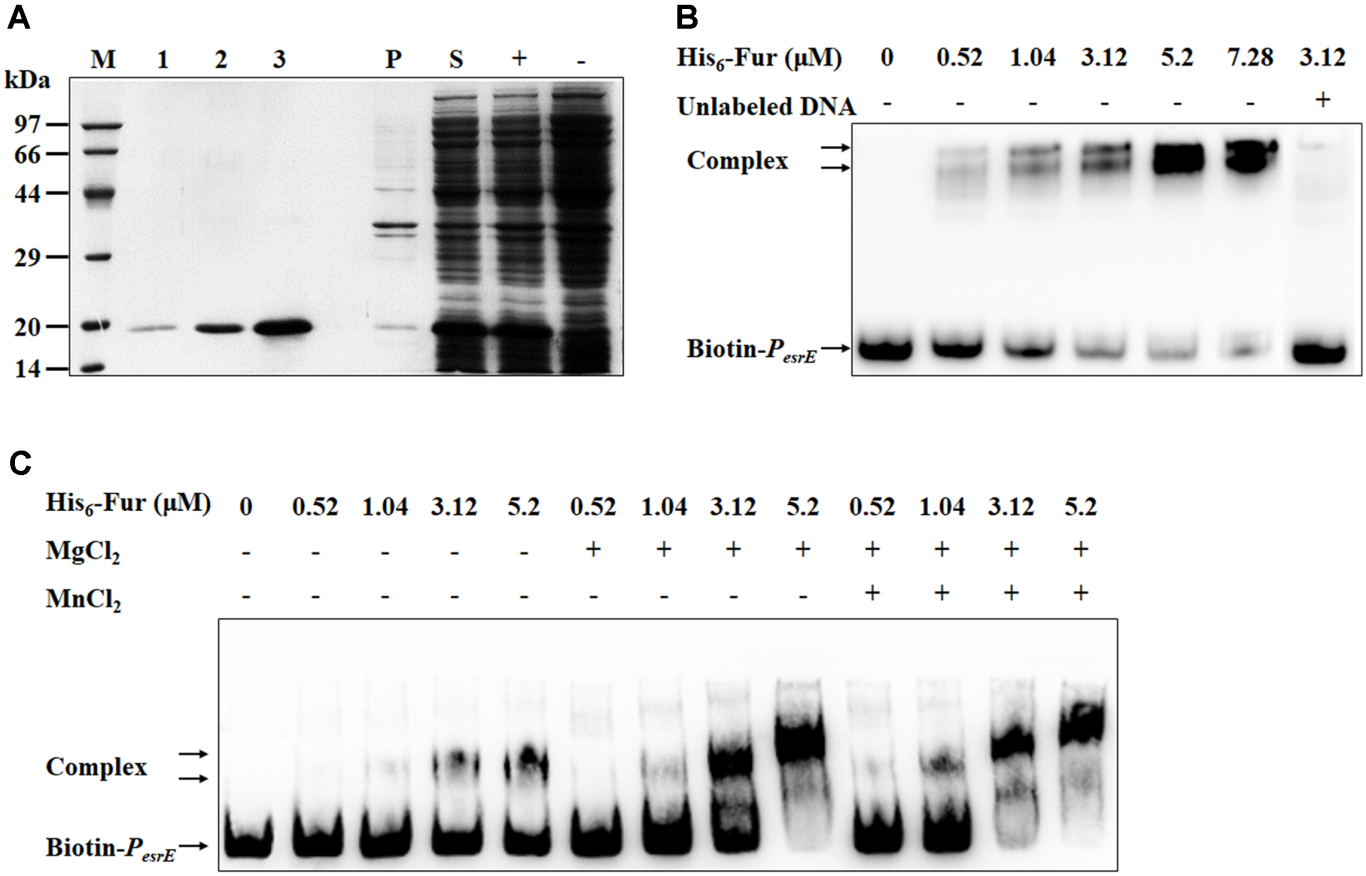
Binding of His_6_-Fur to the promoter region of *esrE*. (**A**) Expression and purification of His_6_-Fur proteins. 1, 2, and 3 indicated proteins eluted by 100, 150, and 200 mM imidazole solution respectively. P indicated precipitation after ultrasonication, S indicated supernatant after ultrasonication, + indicated total proteins from cells induction, and - indicated total proteins from cells without induction. (**B**) EMSA analysis of His_6_-Fur to P_*esrE*_. Biotin-labeled probes Biotin-P_*esrE*_ (188 bp, 10 ng) were incubated with increasing concentrations of purified His_6_-Fur (0, 0.52, 1.04, 3.12, 5.20, and 7.28 μM). 100-fold excess of unlabeled specific competitor P_*esrE*_ was added as control to confirm the specificity of the band shifts. The free probes and DNA-protein complexes are indicated by arrows. (**C**) EMSA analysis of His_6_-Fur to P_*esrE*_ under different conditions. The concentrations of Mg^2+^ and Mn^2+^ used in EMSA were 1 mM and 0.1 mM, respectively.

We first screened the 7 potential proteins by performing β-galactosidase activity assays, and the data showed that these proteins do not regulate the promoter P_*esrE*_ in vivo (Fig. S1). Among these candidate regulators, Fur is a global transcriptional regulator found in most bacteria. Considering the regulation of Fur may need cofactors, thus we chose Fur for further study. To confirm the DNA-binding activity of Fur to P_*esrE*_, His_6_-Fur protein (19-kDa) was overexpressed, purified, and analyzed by SDS-PAGE (Fig. 3A). Then EMSA analysis was carried out using the purified His_6_-Fur with the DNA probe Biotin-P_*esrE*_ in the presence of Mg^2+^ and Mn^2+^. The data showed that His_6_-Fur could bind to Biotin-P_*esrE*_ and generate significantly shifted bands in a concentration-dependent manner (Fig. 3B). The DNA-binding specificity was evaluated by the addition of excess unlabeled specific probe (P_*esrE*_), in which the shifted bands disappeared (Fig. 3B, the last lane), suggesting that His_6_-Fur specifically binds to P_*esrE*_ in vitro. Furthermore, to investigate the effects of Mg^2+^ and Mn^2+^ on the DNA-binding of Fur, EMSAs under different conditions were performed. The results showed that Fur can bind to P_*esrE*_ without divalent metal ions, while Mg^2+^ can facilitate the binding of Fur and P_*esrE*_ (Fig. 3C). These data are consistent with the results of the pull-down assays, indicating that Fur can directly bind to the promoter region of the *esrE* gene.

### Fur Positively Regulates the P_*esrE*_ Promoter when the Cofactor Fe^2+^ Is Present

**Fig. 4:**
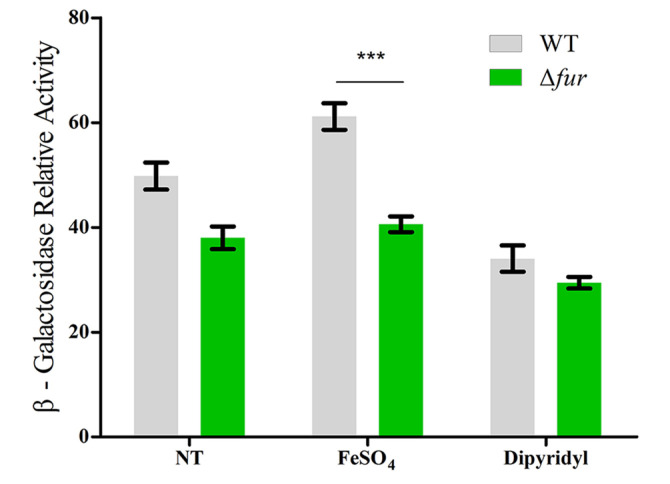
Reporter assays of the effect of Fur on the activity of the P_*esrE*_ promoter under different conditions. NT showed no additive was added. FeSO_4_ showed Fe^2+^ (100 μM) was added. Dipyridyl showed iron-chelator dipyridyl (100 μM) was added, but Fe^2+^ was not added. WT, wild-type strain JM83, Δ*fur*, *fur* inactivation strain. Bars correspond to the mean ± SD of three biological replicates, ***p < 0.001.

To further define the effect of Fur on the activity of the P_*esrE*_ promoter in vivo, we constructed a Fur mutant Δ*fur* in which the internal region of the *fur* gene was replaced by a apramycin resistance cassette. Then the P_*esrE*_ fragment was amplified and fused to the *lacZ* gene to construct reporter plasmid, which was introduced into both the wild-type strain JM83 and the mutant Δ*fur* respectively. Subsequent reporter assays were carried out under different culture conditions, in which Fe^2+^ or the iron-chelator dipyridyl was added or not [38]. The results showed that there was no significant difference in the β-galactosidase relative activity between JM83 and Δ*fur* when iron-chelator dipyridyl was added, which is consistent with the result without any additive (Fig. 4). However, the β-galactosidase relative activity was lower in Δ*fur* compared to JM83 when Fe^2+^ was added, indicating that Fur activates the P_*esrE*_ promoter when the cofactor Fe^2+^ is present.

### Inactivation of the *fur* Gene Inhibits the Growth of *E. coli*

**Fig. 5:**
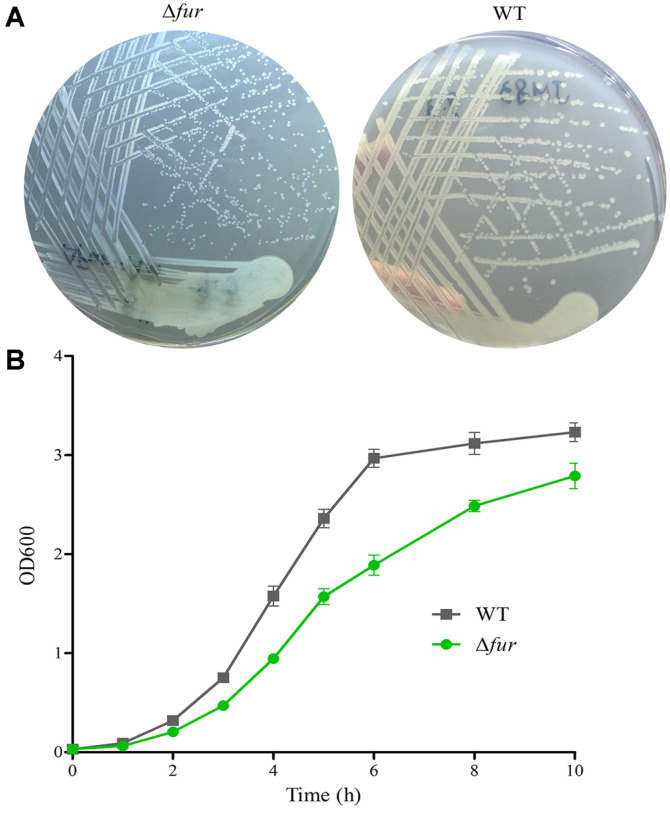
Cell growth analysis of *fur* inactivation strain. Wild-type (WT) and Δ*fur* strains were grown on solid LB plates (**A**) and liquid LB medium (**B**) respectively. The experiments were performed at least 3 times, and the identical patterns were represent.

It has been shown that Fur functions as a global regulator, which is involved in many cellular processes. In addition, our previous studies demonstrated that EsrE sRNA, the target of Fur, is required for aerobic growth of *E. coli* [29]. In view of this, we investigated the effect of Fur on cell growth of JM83. Both strains JM83 and Δ*fur* were cultured and analyzed on solid LB medium and in liquid LB medium respectively. The data revealed that Δ*fur* mutant forms smaller colonies than the wild-type strain JM83 (Fig. 5A), meanwhile, the mutant exhibits retarded growth compared to the wild-type JM83 in liquid LB medium (Fig. 5B), indicating deletion of the *fur* gene also affects cell growth of JM83.

## Discussion

In our previous study, we have shown that expression of EsrE sRNA is under the control of its own promoter [28, 29]. Here, we performed further studies to analyze the regulatory mechanism of EsrE sRNA expression and identify the corresponding *trans*-regulatory elements.

The transcriptional factor Fur has been widely researched in recent years, and functions as a global transcriptional regulator that plays an important role in modulating expression of the genes involved in iron uptake, oxidative stresses, biofilm formation and virulence in various species [39-42]. Fur can function as a repressor which inhibits the binding of the RNA polymerase holoenzyme (RNAP), and also function as an activator through sRNA regulation, RNAP recruitment or antirepressor mechanism [43]. It has been shown that Fur can regulate a number of genes through the regulation of sRNA at the posttranscriptional level. For instance, Fur represses the expression of RyhB sRNA, which downregulates at least six mRNAs encoding iron-binding proteins, including *sdhCDAB* operon encoding succinate dehydrogenase in *E. coli* [44]. Moreover, *sdhCDAB* is regulated by NrrF sRNA as well, the expression of which is controlled by Fur in the human pathogen *Neisseria meningitidis* [45]. Thus, this phenomenon is common in the regulatory network of bacteria, showing that bacteria can regulate the expression of genes in cells layer by layer, and control their metabolism reasonably and effectively. Previously, we showed that EsrE sRNA upregulates the expression of *sdhD* and is required for succinate dehydrogenase activity in *E. coli* [29]. Here, we demonstrated that Fur positively regulates the expression of EsrE sRNA by binding to the promoter region of *esrE* directly (Figs. 3 and 4), indicating that a similar phenomenon of cascade regulation also exists in Fur, EsrE sRNA and *sdhCDAB*. In addition, the recognition mechanism of Fur to the targets has been found conserved in distantly related species, such as *E. coli*, *Pseudomonas aeruginosa* and *Bacillus subtilis*, and the binding sites of Fur are AT-rich boxes (Fur box) [46]. However, although the promoter region of *esrE* contains several AT-rich motifs (Fig. 1A), it lacks a typical Fur box. Thus, further studies will be performed to reveal the explicit relationships and the specific mechanisms among Fur, EsrE sRNA, *sdhCDAB* and the corresponding cell phenotype.

In addition, the results of β-galactosidase activity assay showed that at least one negative and one positive *cis*-regulatory element are involved in transcriptional regulation of the *esrE* promoter (Fig. 1C), indicating there may be one or more corresponding *trans*-regulatory elements. Accordingly, pull-down assays illustrated that there are several candidate P_*esrE*_-interactive regulators besides Fur (Fig. 2 and Table 3). These data demonstrated that the regulatory mechanism of EsrE sRNA expression is complicated. In subsequent studies, we will continue to analyze the other regulators and the physiological signals to which EsrE sRNA responds.

In conclusion, this report demonstrated that Fur regulates EsrE sRNA expression and shed light on the regulation of EsrE for the first time. Our results elaborate the relationship among Fur, EsrE sRNA, and *sdhCDAB* operon, and thus contribute to the illustration of the ecological behavior of the bacteria.

## Supplemental Materials



Supplementary data for this paper are available on-line only at http://jmb.or.kr.

## Figures and Tables

**Table 1:** Strains and plasmids used in this study.

Strains or plasmids	Genotype and/or description	Source or reference
Strains		
*E. coli*		
JM83	F’, ara, Δ(*lac-pro* AB), *rpsL*, (Str^r^), Φ80, *lacZ*ΔM15	
BL21 (DE3)	F^-^ *ompT* *hsdS* *gal* *dcm*	Novagen
Δ*fur*	JM83, in-frame deletion in the *fur* gene, Am^r^	This study
Plasmids		
pSPORT1	Plasmid vector, Ap^r^, *lacZ*^-^	Stratagene
pSP-Z	3.1 kb *Pst*I/*Bam*HI fragment containing the *lacZ* gene in pSPORT1, Ap^r^	This study
pSZ1	pSP-Z carrying the *lacZ* reporter gene controlled by S1V3	This study
pPZ40	pSP-Z carrying the *lacZ* reporter gene controlled by P40V3	This study
pPZ42	pSP-Z carrying the *lacZ* reporter gene controlled by P42V3	This study
pPZ43	pSP-Z carrying the *lacZ* reporter gene controlled by P43V3	This study
pET-28a (+)	*E. coli* expression vector	Novagen

**Table 2:** Primers used in this study.

Primers	Sequence(5' to 3')
Construction of the recombinant proteins	
S1	CCCAAGCTTTGCACCGTTATCGCCTACGCCAGTG
P40	GAAGCTTACCGCACTGATTCG
P42	CCCAAGCTTTGCAGGGCGATATTCAG
P43	CCCAAGCTTGTGGTGCAAAACTTCG
V3	GATTCCTCCATGGGCGATATCACCG
PD*-1	Biotin-TGCACCGTTATCGCCTACGC
PD-1	TGCACCGTTATCGCCTACGC
PD-2	GCGATATCACCGGTATAAGG
DZ*-1	Biotin-GCAAAGCCATGCGCGGAGGC
DZ-1	GCAAAGCCATGCGCGGAGGC
DZ-2	CAGTTTTTCCAGCCGTTTGG
Fur-1	CGCCATATGATGACTGATAACAATACC
Fur-2	CCGGAATTCTTATTTGCCTTCGTGCG
fur-QC1	ATGACTGATAACAATACCGCCCTAAAGAAAGCTGGCCTGATTCCGGGGATCCGTCGACC
fur-QC2	TTATTTGCCTTCGTGCGCATGTTCATCTTCGCGGCAATCGTGTAGGCTGGAGCTGCTTC
fur-JD1	TTGCCAGGGACTTGTGGT
fur-JD2	CTGGCAGGAAATACGCAG

**Table 3:** Candidate DNA-binding proteins identified by DNA affinity chromatography and mass spectrometry.

Protein	Accession Number	Protein description	MW/kDa
GntR	YP_026222.1	D-gluconate inducible gluconate regulon transcriptional repressor	36
RdgC	NP_414927.1	Nucleoid-associated ssDNA and dsDNA binding protein	34
UidR	NP_416135.1	DNA-binding transcriptional repressor	22
SeqA	NP_415213.1	Negative modulator of initiation of replication	20
Dps	NP_415333.1	Stress-inducible DNA-binding protein	19
DicA	NP_416088.1	Qin prophage predicted regulator for DicB	17
Fur	NP_415209.1	Ferric uptake regulation protein	17
