# Supplementary material for: sRNA EsrE Is Transcriptionally Regulated by the Ferric Uptake Regulator Fur in Escherichia coli
Source: J Microbiol Biotechnol. 2019 Nov 6;30(1):127–35. doi: 10.4014/jmb.1907.07026 (PMC9728176; doi:10.4014/jmb.1907.07026)
Supplement: Supplementary file 1 [file JMB-30-1-127-supple.pdf]

J. Microbiol. Biotechnol.
https://doi.org/10.4014/jmb.1907.07026

jmb

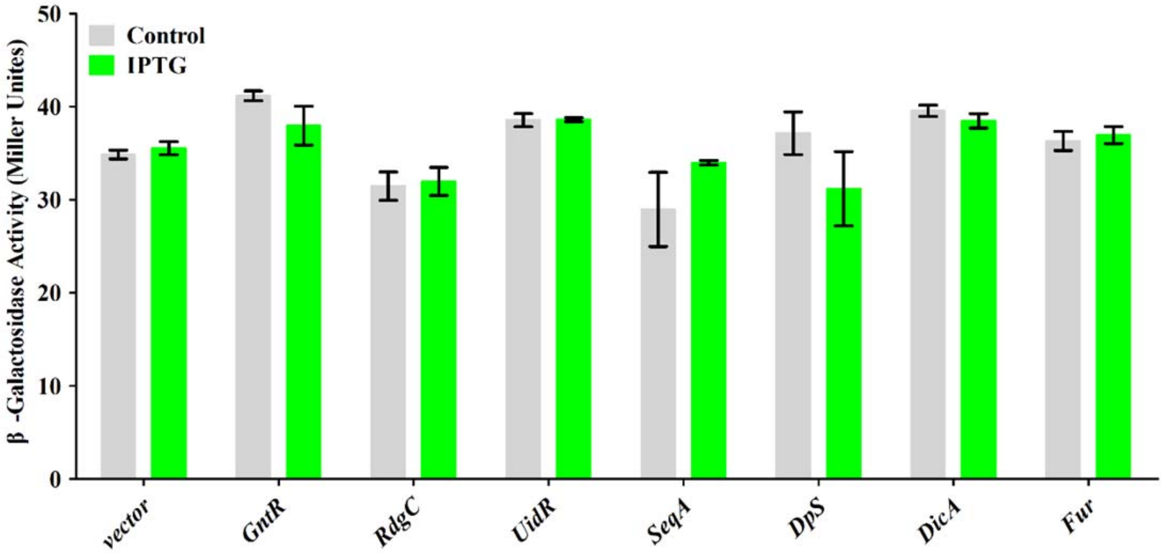

Figure S1 Reporter assays of the effect of potential proteins on the activity of the P_{esrE} promoter.

Table S1 The obtained peptide mass patterns analyzed by MASCOT 2.2 software

Protein	No.	Reference	Pep Count	Unique Pep Count	Cover Percent (%)
GntR	1	regulator GntR OS=Escherichia coli (strain K12) GN=gntR PE=1 SV=1	1	1	2.42
	2	tr|A0A0D6K793|A0A0D6K793_ECOLI DNA-binding transcriptional repressor OS=Escherichia coli (strain K12) GN=gntR PE=4 SV=1	1	1	2.42
RdgC	1	sp|B1XEY2|RDGC_ECODH Recombination-associated protein RdgC OS=Escherichia coli (strain K12 /DH10B) GN=rdgC PE=3 SV=1	12	12	32.34
	2	sp|C4ZTF1|RDGC_ECOBW Recombination-associated protein RdgC OS=Escherichia coli (strain K12 / MC4100 / BW2952) GN=rdgC PE=3 SV=1	12	12	32.34
	3	tr|A0A0D6JYH1|A0A0D6JYH1_ECOLI Nucleoid-associated ssDNA and dsDNA binding protein competitive inhibitor of RecA function OS=Escherichia coli (strain K12) GN=rdgC PE=4 SV=1	12	12	32.34
	4	sp|P36767|RDGC_ECOLI Recombination-associated protein RdgC OS=Escherichia coli (strain K12) GN=rdgC PE=1 SV=1	12	12	32.34
UidR	1	tr|A0A0D6K2E9|A0A0D6K2E9_ECOLI DNA-binding transcriptional repressor OS=Escherichia coli (strain K12) GN=uidR PE=4 SV=1	1	1	5.61
	2	sp|P0ACT6|UIDR_ECOLI HTH-type transcriptional regulator UidR OS=Escherichia coli (strain K12) GN=uidR PE=3 SV=1	1	1	5.61
SeqA	1	sp|P0AFY8|SEQA_ECOLI Negative modulator of initiation of replication OS=Escherichia coli (strain K12) GN=seqA PE=1 SV=1	96	9	54.70
	2	tr|A0A0D6K004|A0A0D6K004_ECOLI Regulatory protein for replication initiation OS=Escherichia coli (strain K12) GN=seqA PE=4 SV=1	96	9	54.70
Dps	1	sp|B1X7E2|DPS_ECODH DNA protection during starvation protein OS=Escherichia coli (strain K12 / DH10B) GN=dps PE=3 SV=1	5	4	31.74

	2	tr|A0A0D6K0D7|A0A0D6K0D7_ECOLI Fe-binding and storage protein stress-inducible DNA-binding protetin OS=Escherichia coli (strain K12) GN=dps PE=4 SV=1	5	4	31.74
	3	sp|P0ABT2|DPS_ECOLI DNA protection during starvation protein OS=Escherichia coli (strain K12) GN=dps PE=1 SV=2	5	4	31.74
	4	sp|C4ZXY4|DPS_ECOBW DNA protection during starvation protein OS=Escherichia coli (strain K12 / MC4100 / BW2952) GN=dps PE=3 SV=1	5	4	31.74
DicA	1	tr|A0A0D6K1T0|A0A0D6K1T0_ECOLI Qin prophage predicted regulator for DicB OS=Escherichia coli (strain K12) GN=dicA PE=4 SV=1	1	1	5.19
	2	sp|P06966|DICA_ECOLI HTH-type transcriptional regulator DicA OS=Escherichia coli (strain K12) GN=dicA PE=3 SV=1	1	1	5.19
Fur	1	tr|A0A0D6JZZ9|A0A0D6JZZ9_ECOLI DNA-binding transcriptional dual regulator of siderophore biosynthesis and transport OS=Escherichia coli (strain K12) GN=fur PE=4 SV=1	1	1	8.78
	2	sp|P0A9A9|FUR_ECOLI Ferric uptake regulation protein OS=Escherichia coli (strain K12) GN=fur PE=1 SV=1	1	1	8.78
